# Role of Key Micronutrients from Nutrigenetic and Nutrigenomic Perspectives in Cancer Prevention

**DOI:** 10.3390/medicina55060283

**Published:** 2019-06-18

**Authors:** Alexandra Iulia Irimie, Cornelia Braicu, Sergiu Pasca, Lorand Magdo, Diana Gulei, Roxana Cojocneanu, Cristina Ciocan, Andrei Olariu, Ovidiu Coza, Ioana Berindan-Neagoe

**Affiliations:** 1 Department of Prosthetic Dentistry and Dental Materials, Division Dental Propaedeutics, Aesthetic, Iuliu Hatieganu University of Medicine and Pharmacy, Cluj-Napoca, 23 Marinescu Street, 40015 Cluj-Napoca, Romania; irimie.alexandra@umfcluj.ro; 2 Research Center for Functional Genomics and Translational Medicine, Iuliu Hatieganu University of Medicine and Pharmacy, 23 Marinescu Street, 40015 Cluj-Napoca, Romania; cornelia.braicu@umfcluj.ro (C.B.); pasca.sergiu123@gmail.com (S.P.); lorand.magdo@gmail.com (L.M.); cojocneanur@gmail.com (R.C.); ioana.neagoe@umfcluj.ro (I.B.-N.); 3 MEDFUTURE-Research Center for Advanced Medicine, University of Medicine and Pharmacy Iuliu Hatieganu, 23 Marinescu Street, 40015 Cluj-Napoca, Romania; diana.c.gulei@gmail.com (D.G.); crisciocan@gmail.com (C.C.); 4 Nordlogic Software, 10–12, Rene Descartes Street 400486 Cluj-Napoca, Romania; andrei.olariu@nordlogic.com; 5 Department of Radiotherapy with High Energies and Brachytherapy, Oncology Institute “Prof. Dr. Ion Chiricuta”, Street Republicii, No. 34–36, 400015 Cluj-Napoca, Romania; 6 Department of Radiotherapy and Medical Oncology, “Iuliu Hatieganu” University of Medicine and Pharmacy, Street Louis Pasteur, No. 4, 400349 Cluj-Napoca, Romania; 7 Department of Functional Genomics and Experimental Pathology, “Prof. Dr. Ion Chiricuta” The Oncology Institute, 34-36 Republicii Street, 400015 Cluj-Napoca, Romania

**Keywords:** nutrigenetics, nutrigenomics, cancer, chemoprevention

## Abstract

Regarding cancer as a genetic multi-factorial disease, a number of aspects need to be investigated and analyzed in terms of cancer’s predisposition, development and prognosis. One of these multi-dimensional factors, which has gained increased attention in the oncological field due to its unelucidated role in risk assessment for cancer, is diet. Moreover, as studies advance, a clearer connection between diet and the molecular alteration of patients is becoming identifiable and quantifiable, thereby replacing the old general view associating specific phenotypical changes with the differential intake of nutrients. Respectively, there are two major fields concentrated on the interrelation between genome and diet: nutrigenetics and nutrigenomics. Nutrigenetics studies the effects of nutrition at the gene level, whereas nutrigenomics studies the effect of nutrients on genome and transcriptome patterns. By precisely evaluating the interaction between the genomic profile of patients and their nutrient intake, it is possible to envision a concept of personalized medicine encompassing nutrition and health care. The list of nutrients that could have an inhibitory effect on cancer development is quite extensive, with evidence in the scientific literature. The administration of these nutrients showed significant results in vitro and in vivo regarding cancer inhibition, although more studies regarding administration in effective doses in actual patients need to be done.

## 1. Introduction

Cancer should not be considered as a single disease, but as a multitude of different genetic (irreversible) and epigenetic alterations (reversible in some situations) that act in tandem, mirrored by changes in patterns exhibited in the transcriptome [1]. Genetics, simply put, investigates genes, genetic variation and heredity in organisms. The word “epigenetics” literally means “above genetics” and, thus, refers to all the mechanisms that control or regulate gene expression without actually changing the DNA sequence. This means that epigenetic changes encompass all molecular modifications to DNA or chromatin. The most frequent and extensively investigated epigenetic modification that happens post-translationally is DNA methylation [2]. Examples of genes with some “epigenetic” memory of early life experiences are those related to energy acquisition, storage and use. One such example is leptin, involved in the development of obesity. It encodes a hormone that specifically regulates energy intake and expenditure. It has been proposed that epigenetic variants of leptin could explain the phenomena of low plasma concentrations. More specifically, the promoter region of leptin can be methylated in somatic tissues of humans and, thus, demonstrates epigenetic variation [2].

There is great interest in investigating the relationships between the predisposition for different cancers, their associated prognosis and exposure to different risk factors like diet. This inquiry is based on the fact that bioactive agents within daily nutrients hold great promise in oncology [3] because of their capacity to regulate coding or non-coding genes [4,5] and as adjuvant support for cancer therapy [6].

Nutrigenetics studies nutrition at the gene level, focusing on the way that certain gene variants can influence and are influenced upon by their interaction with nutrients. Nutrigenomics, on the other hand, studies the effects of nutrients on genomic and transcriptomic profiles, and their subsequent consequences on the proteome and metabolome [7]. By predicting the functional interactions between nutrients and genomes, the emerging and developing field of personalized medicine can incorporate nutrition, facilitating the step forward toward personalized cancer therapy. This is based on the capacity of certain nutrients to specifically activate cancer inhibitory mechanisms, thereby targeting important hallmarks of cancer like apoptosis or the impairment of angiogenesis [8,9,10].

The aim of this review is to evaluate and present the effects that some key micronutrient components (vitamin A, vitamin C, vitamin D and Selenium) and some macronutrients (polyunsaturated fatty acids, prebiotics and probiotics) can have in the prevention and/or therapy of different cancer types. After all, one function of personalized medicine is the identification of critical interactions in the cancer–diet relationship specific to the patient and their genome. The nutrients were chosen based on the in vitro or in vivo experimental data available, specifically ensuring that there was an association between the nutrient and a molecular pathway or gene. Furthermore, we wanted to discuss nutrients that are readily accessible and have been well documented.

As an effect, there are future practical applications regarding personalized nutrition. This promising approach characterizes the genetic variants of each individual, monitoring how they react to a diet in light of the specific personalized nutrient intake. Based on these individual investigations, each person could receive a diet validated to give the optimal results in concordance with their genomic background. The current technologies/techniques used for the study of nutrigenetics and nutrigenomics are shown in Table 1. The demand for determining genome, transcriptome, proteome, metabolome and mutation-specific profile characteristics has led to the implementation of several technologies, some of which are simple and inexpensive technologies, such as polymerase chain reaction (PCR)-based methods [7,11,12,13]. The more complex and costly technologies consist of microarray, Sanger sequencing and next generation sequencing (NGS), mass spectrometry (MS), and liquid chromatography coupled with mass spectrometry (LC–MS). They offer more comprehensive information, but are not available on a wide scale. At the same time, the high amount of raw data generated requires specialized bioinformatic analyses executed by specific software and performed in an informed manner by a highly trained bioinformatician [7,14].

## 2. Cancer Risk Represents a Sum of Complex Interactions of Environmental Exposures

Cancer risk represents the synergy of complex interactions encompassing the exposure to different environmental factors, hereditary genetic alterations and epigenetic modifications. These events are accumulated during genotoxic alterations, as a response to environmental damage [37]. Hereditary cancers account for 5–10% of all cancers; the remaining malignancies can be caused by somatic mutations with consequences of environmental exposure exhibited at the expression level for coding and non-coding genes [38,39]. Therefore, a chemopreventive and therapeutic effect can be achieved by specifically increasing the concentration of a compound, retrieved naturally from a normal diet in functional foods or in an enriched form as nutraceuticals [40].

As previously mentioned, there are two strategies that could offer important missing information, thereby linking environmental exposure to intrinsic cancer risk: nutrigenetics and nutrigenomics. Nutrigenetics makes the connection between the human genome, nutrition and exposure, with the gene as the focal point. It has the potential to be exploited for personalized diets, preserving the health state of an individual, preventing the onset of diseases and lastly for adjuvant treatment. The field of nutrigenomics gives a more integrated view of how nutrients effect various gene expressions and, implicitly, the transcript profiles relating to those genes, with direct effect exhibited in proteomic and metabolomic activities [41,42]. This field of study was conceived on the assumption that nutrients can influence gene expression by acting directly on the genome [43], or indirectly by means of epigenetic mechanisms. Also, nutrients appear to be able to influence different cellular processes [9], some of which are related to tumorigenesis [43]; therefore, one consideration is how certain nutrients have an influence on cancer development or progression [44,45]. Natural nutrients are able to disrupt tumorigenesis at multiple ‘omic’ levels and, concurrently, increase the chemotherapeutic efficacy and reduce the side effects related to these treatments [46]. One of the relevant examples is related to oral cancer, which can be arguably prevented by maintaining good oral hygiene, eliminating the use of tobacco and alcohol products, and by having a balanced healthy diet. All these have protective effects and can decrease the risk of oral cancer, in which environmental exposure has the most important role [47]. The protective effects of a diet rich in vegetables and fruits were demonstrated to reduce the risk of oral cavity and oropharynx malignancies in a Spanish patient cohort, especially among smokers and alcohol drinkers (patients with an already increased risk for oral cancer) [48].

It is now well established that one of the risks for cancer development consists in improper diet, which contains an increasing amount of processed foods and high sugar levels, all potentially acting as malignant drivers. Apart from this, it has been assumed by some studies that several nutrients or specific dietary components are able to decrease the possibility of malignant cell transformation; or, moreover, to inhibit the growth and spread of pre-existing malignant masses [49,50]. Even if many in vitro studies have shown that specific components from the everyday diet can act as cancer inhibitors, there is still no clear evidence regarding the pro- or anti-carcinogenic characteristics of nutrients. In spite of the large amount of preclinical studies and clinical trials, most of them present only a borderline-significant effect [51].

**Figure 1:**
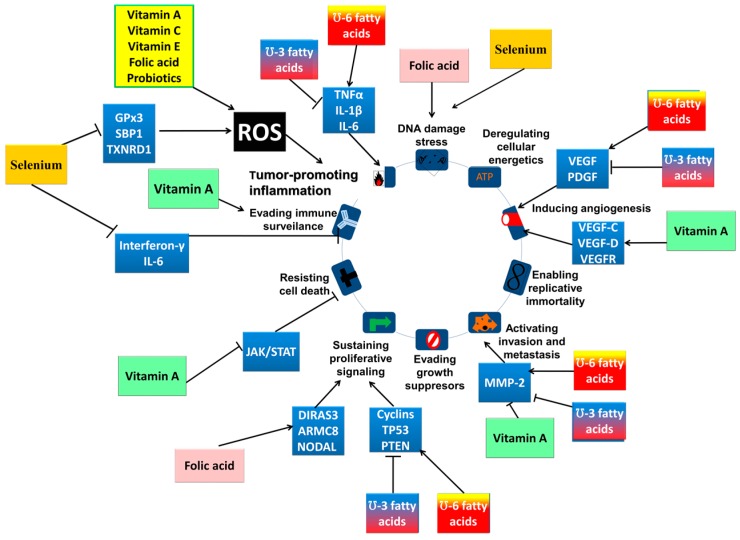
Nutrients’ molecular targets and their intermediaries associated with the hallmarks of cancer. The major impact of nutrients is through their action on reactive oxygen species (ROS) production, which has a critical role in tumor-promoting inflammation. Aside from this effect, nutrients have been shown to effect multiple hallmarks of cancer: for example, fatty acids act on tumor-promoting inflammation, the induction of angiogenesis, the activation of invasion and metastasis and the sustenance of proliferative signaling. Other effects can be observed, significantly impacting on a person’s cancer susceptibility and prognosis, aspects of which can be modulated by patient diet in a directed manner, leading to the development of personalized nutrition.

To determine the influence of nutrients on cancer, they can be cross-linked with the hallmarks of cancer through their molecular intermediaries. The most affected cancer hallmark is tumor-promoted inflammation through oxidative stress caused by reactive oxygen species [52]. The most relevant data are summarized in Table 2 and Figure 1.

Nutrition can be beneficial or detrimental, depending on the person’s genetic profile and variation. An example would be the case of coffee consumption, where certain single nucleotide polymorphisms (SNPs) for alleles identified for GCKR, MLXIPL, BDNF and CYP1A2 could be connected with an excessive intake of coffee; interestingly, these same alleles were initially linked to smoking, adiposity and fasting levels of insulin or glucose [96].

## 3. Vitamins

### 3.1. Vitamin C

Among the popular vitamins that are widely available in natural fruit or supplement form, vitamin C or ascorbic acid is the most commonly known and taken. Vitamin C concentrations from the plasma of cancer patients were significantly reduced when compared to healthy controls, raising several questions related to cancer and vitamin C involvement [49]. To counteract the growth of a malignant tumor mass, Vitamin C can be administered for its dose-dependent anti-carcinogenic properties [40]. The reported dose-dependent effects of Vitamin C are also specific to cancer type; for example in melanoma, high doses of vitamin C induced apoptosis, whereas low doses promoted cell proliferation [55,97]. However, it should be noted that even high doses of Vitamin C are not effective against malignant disease. Additionally, there are some unwanted side effects caused by high dose accumulation in normal cells, which can be harmful due to pro-oxidant action, whose effects are observed at millimolar concentrations [40]. One of the aforementioned anti-carcinogenic properties attributed to Vitamin C is sensitivity to chemotherapy [98]. Another anti-carcinogenic property comes from its function as an anti-oxidant; ascorbic acid produces small amounts of hydrogen peroxide. The hydrogen peroxide quantities generated from high doses of Vitamin C can be lethal to cancer cells due to their low amounts of hydrogen peroxide-processing enzymatic and non-enzymatic mechanisms. The accumulation of hydrogen peroxide, through the induction of apoptosis, can eventually lead to tumor cell lysis [40,49]. A case in point is found in human tongue carcinoma cells, where high doses of vitamin C induced anti-tumor effects via the generation of hydrogen peroxide and superoxide anion radicals [62]. In another study carried out on laryngeal squamous cell carcinoma, vitamin C was revealed to activate necrotic cell death mechanisms via ROS (reactive oxygen species) production and the stimulation of protein kinase C (PKC) signaling, causing increased cytosolic calcium [99]. It has been established that mice receiving intravenous administration can reach cytotoxic concentrations of vitamin C, similar to the results obtained in vitro [100,101].

Dietary vitamin C is generally transferred by two transporter proteins that carry this molecule across cell membranes and modulate oxidative stress: sodium-dependent vitamin C transporter (SVCT) and glucose transporter (GLUT). Moreover, oxidative stress is influenced by the antioxidant enzymes manganese super oxide dismutase (MnSOD), glutathione S-transferase (GS), and haptoglobin (Hp)—a protein linked to hemoglobin. The *Hp* gene encodes two structurally different alleles: Hp1 and Hp2. It is this Hp2-2 genotype, observed in 48% of Caucasians and 52% of Asians, that is associated with vitamin C deficiency [58]. Oxidative stress, in which vitamin C has long been known to be involved, has an effect on apoptosis through regulating Bcl-2, a known anti-apoptotic protein [102]. Aside from the participation of vitamin C in oxidative stress, it has been shown that this active substance inhibits the formation of N-nitrosamine carcinogenic compounds [103] and modulates immune response [104]. These modulatory mechanisms may explain the inverse relationship between the variation in the quantity of ascorbic acid ingested and its effects, as described, on different cancer types: lung, stomach, larynx, breast, colon, head and neck carcinoma [61,62,99,105]. Vitamin C intake does not only have an effect on cancer prevention. It also has an impact on cancer-related mortality in breast cancer [106], and lowers the necessary doses of chemotherapeutic agent to achieve comparative treatment effects [107]. All things considered, the therapeutic role of vitamin C has begun to be more thoroughly investigated.

A beneficial effect was observed in the case of short-term diet supplementation of vitamin E and C complexes against radiotherapy-induced xerostomia in head and neck cancer [61]. In addition, another protective-like effect from dietary vitamin C intake was observed in a patient cohort of forty-one men with squamous cell oral or pharyngeal cancer, when compared to 398 male healthy control subjects [108]. In conclusion, epidemiological studies revealed that vitamin C can reduce the risk of malignancies [109,110]. However, one must not forget that the beneficial effects of vitamin C cannot be separated from the beneficial effects of a healthy diet rich in fruits or vegetables [108,110].

### 3.2. Vitamin A

Dietary vitamin A is a product derived from a variety of carotenoids found in plants, with a broad range of beneficial effects on human health. It not only acts as an antioxidant, protecting against oxidative stress and DNA damage, but also at the cellular level, it modulates cell growth while regulating methylation. Vitamin A is considered to have a more complex mechanism of action that is currently being investigated [53], consisting in a wide range of biochemical and immunological roles against cancer [111]. For example, a study revealed that vitamin A reduced oral mucositis, a consequence of chemotherapy [112]. Vitamin A or its related analogs, the retinoids, were demonstrated to have the capacity to reduce head, neck and lung carcinogenesis in animal models [113]. The inhibition of premalignant lesion was demonstrated to be achieved via the regulation of genes involved in cell growth and differentiation. Retinoids and lycopene can have beneficial effects in treating oral leukopathia, with important roles in oral cancer prevention [114]. A combination of bexarotene and retinoids was able to reduce the chemical induction of oral carcinogenesis by 4-nitroquinoline 1-oxide, via a mechanism of ROS prevention [115].

Retinoic acid amide has been shown to inhibit the JAK-STAT pathway in lung cancer, leading to apoptosis [116]. Vitamin A-associated effects are completed mainly via all trans retinoic acid (ATRA), which targets a wide range of nuclear receptors. These nuclear receptors include retinoic acid receptor (RAR), retinoid X receptor (RXR), and peroxisome proliferator-activated receptor (PPARβ/δ), where polymorphic retinoic acid (RA) response elements are able to activate the kinase cascades (assimilated in the nucleus via the phosphorylation of RA signaling effectors) [54]. The nuclear receptors targeted by ATRA have been shown to have a role in oral cancer [117]. Therefore, ATRA treatment was able to restore gap junctional intercellular communication for oral cancer cells by the upregulation of Cx32 and Cx43 [117].

RAR promoter methylation can be used as a predictive diagnostic marker for non-small cell lung cancer (NSCLC) [118]. The hypermethylation of RAR promoter has been shown to be associated with other known factors that influence lung cancer, one of the most important being cigarette smoke [119]. The therapeutic induced hypomethylation of RAR promoter has been achieved by using curcumin, thus identifying a possible anti-cancer therapy [120]. In addition, retinoid X receptor (RXR) and histone deacetylase (HDAC) have been in vitro and in vivo targeted for activation and inhibition, respectively, revealing pleiotropic antitumor activities [121]. The repression of PPAR has been shown to promote chemoresistance in NSCLC [122], while PPAR agonists have been associated with a role in preventing and treating lung cancer [123]. PPAR-related mechanisms have been used in experimental models to inhibit key genes involved in tumorigenesis, such as matrix metalloproteinase 2 (MMP-2) in the lung adenocarcinoma cell line A549 [124]. Some tumors were observed to be resistant to the antiproliferative action of RA, mainly via protein kinase B (AKT) or different mitogen activated protein kinases (MAPKs), including extracellular signal-regulated kinase (ERK), Jun N terminal kinase (JNK) or p38 [55]. Despite the exposition of the possible underlying molecular mechanisms, the association between vitamin A (including retinol and carotenoids) and cancer still remains controversial.

A meta-analysis study demonstrated that dietary intake of vitamin A, beta-carotene and lycopene is inversely associated with pancreatic cancer [125]. On the contrary, there are also studies showing that an increase in vitamin A dietary intake is linked to an increase in cancer incidence. Several studies grouped together in a meta-analysis showed a slight increase in cancer incidence simultaneous with vitamin A consumption, when compared to the majority of β-carotene supplements which showed no significant correlation with cancer incidence [126,127]. Similar results are shown in the CARET study (Beta-Carotene and Retinol Efficacy Trial), in which a positive correlation between beta-carotene consumption and lung cancer has been shown [128].

Altogether, these studies show the heterogeneity of cancer susceptibility, especially regarding the link between cancer and vitamin A or beta carotene consumption. Nevertheless, the effect that Vitamin A has on different diseases, including oral cancer, must be considered in correlation with the synthesized metabolized by-products, organism microbiota and interactions with non-provitamin A carotenoids [108].

### 3.3. Vitamin D

Another type of vitamin that has been associated with low risk for cancer development is vitamin D, previously known for its relation to bone metabolism and, through extension, bone diseases. The analysis of heterogeneous population groups in the light of vitamin D status has shown that this molecule holds protective properties, especially in the context of oral, head and neck, breast, ovarian, prostate and colon cancers [129,130].

The dual role of vitamin D in cancer development is dependent on the administrated amount and time [71]. The vitamin D receptor (VDR) is a ligand-inducible transcription factor that targets genes with key roles in cellular processes related to metabolism, inflammation, cell growth and differentiation [131]. It has been demonstrated that genetic polymorphisms of VDR genes and vitamin D metabolism pathway initiators, CYP27B1 and CYP24B1, are related to a specific susceptibility to and patient prognosis of oral squamous cell carcinoma [132]. For example, VDR FokI gene polymorphism was related to an unfavorable survival rate in oral cancer [132]. Vitamin D defective pathway might have an etiologic role in the development of prostate cancer [133], colon and breast malignancies [134].

At the genomic level, vitamin D mediates a wide range of nuclear effects via VDR. Conversely, at the cellular level, the same transcription factors induce a signaling cascade in both the membrane and the cytosol. This fact sustains the complex role of vitamin D in cellular immunity, providing protection against pathogens [135]. In the clinical context, the level of circulating 25OH vitamin D has been shown to be positively correlated with overall survival and progression-free survival [136]. As a therapeutic approach, vitamin D has demonstrated the ability to induce radiosensitization in breast cancer cells [137]; unfortunately, there was only a modest effect in vivo [138]. In pancreatic cancer, the active form of vitamin D and its analogs, through their intermediary effects on p21 and p27, have been shown to induce differentiation, prevent proliferation, and inhibit angiogenesis [139]. Lastly, Vitamin D can prevent apoptosis resistance in oral cancer cells [129] by modulating the VDR expression in precancerous lesions [140].

### 3.4. Folic Acid

Folic acid, or the natural form present in food sources, folate, is now the substrate of an intense debate regarding its pro- or anticarcinogenic effects. Low folate concentrations have been linked to carcinogenesis by the incorporation of uracil in the DNA helix and the causation of double stranded breaks, which in turn can cause cancer-driven mutations [141]. Some controversial literature data showed that in some cases this supplement can inhibit the development of malignant masses, whereas in others it can contribute to the progression of cancer; thus, folate can act as a “double-edged sword”. Folate is an essential water-soluble factor found in food sources. It is one of the nutrients that are widely used in fortification programs, either from natural sources or in synthetic form. This is due to its important role in the processes of DNA, RNA, and protein methylation, as well as DNA synthesis and maintenance [142]. A methylation profiling study in the case of 162 elderly subjects versus 14 controls led to the identification of 431,312 differentially methylated genes. The differentially methylated regions (DMRs) were mainly grouped in six regions, based on comparing the folic acid group versus the control group. An important modification pattern was observed in the case of *DIRAS3*, *ARMC8*, and *NODAL* genes, involved in carcinogenesis and early embryonic development [73].

One important gene implicated in the metabolism of folic acid is methylene tetrahydrofolate reductase (MTHFR), which catalyzes the synthesis of 5-methyl tetrahydrofolate. A significant polymorphism at the level of the *MTHFR* gene is C677T, which induces increased homocysteine concentrations and DNA hypomethylation. Furthermore, it has been shown to be associated with neural tube defects, white matter integrity in Alzheimer patients, venous thrombosis, colorectal cancer survival, breast cancer and leukemia [143,144,145,146,147,148,149]. Continuing on, the links between MTHFR polymorphisms and lung cancer have also been extensively studied. C677T polymorphism is associated with a higher risk of developing this malignancy [150,151,152,153].

Folic acid is involved in physiological processes related to DNA methylation which, once unbalanced, will lead to alterations in DNA biosynthesis, repairing and methylation mechanisms. Perturbing these processes can accelerate aging mechanisms and carcinogenic processes, in addition to affecting normal embryonic development [72,73]. It is clear that this small compound is involved in the genomic stability of eukaryotic cells [154]. It was demonstrated that DNMT3B methylation enzyme polymorphism (C46359T and SHMT1 C1420T) can be involved in the regulation of the folate pathway, related to carcinogenesis in the head and neck [155].

Several studies link folate status to various types of cancer, such as lymphoma, leukemia, colorectal cancer, breast cancer and prostate cancer [156,157,158,159,160]. As an application in lung cancer patients, a variety of folic acid conjugated nanoparticles were developed and showed enhanced antitumor activity [22,160,161,162,163]. Dietary folate and vitamin B6 can have protective roles for nasopharyngeal carcinoma, a fact demonstrated in a large patient cohort on a Chinese population [164], and in an Egyptian patient cohort [165].

As a therapeutic agent, folic acid has been used in various combinations showing modest effects in preventing colorectal cancer [166], or in preventing secondary effects of chemotherapy for lung cancer [167]. Unfortunately, little to no effects have been shown in the prevention of colorectal adenomas [168]. There is a demand for more studies utilizing folic acid as an adjuvant. In these particular situations, one should always remember that cancer is not a single disease, but a heterogeneous combination of pathological states.

## 4. Selenium

Selenium is a natural mineral with powerful effects on the organism, even in small amounts. Selenium enters the food chain through plants; its amount and bioavailability in the soil is typically reflected within plants. Selenium is normally acquired by humans through diet, but may also be derived from drinking water, environmental pollution, and supplementation. RNAseq-based studies led to the identification of 25 selenoproteins, presented as the human selenoproteome, centered on the selenocysteine insertion RNA structures and the coding capacity of UGA codons [169]. This information has been continuously updated through recent research [170,171]. With our improved understanding of the genome, selenium offers new data concerning its significance for human health [76].

Studies have connected genetic variants in selenium metabolism to the progression of complex pathologies like cancer [172]. This essential trace element is a constitutive part of selenocysteine, an essential amino acid that is incorporated in particular proteins like glutathione peroxidases (GPxs) and thioredoxin reductases (TrxRs). Moreover, GPX3 promoter methylation has been shown to have a predictive value in oxaliplatin resistance in colorectal cancer [173].

These selenium-containing proteins possess a wide range of biological functions, from antioxidant to anti-inflammatory activities [74]. There are more than 30 genes that affect selenium uptake, metabolism, and excretion. Selenium plays a central role in the elimination of reactive oxygen species, molecules that, in high doses, can contribute to the malignant phenotypic transformation of cancer cells [74]. Selenium is also important for the recirculation of cancer inhibitory-antioxidants through the body, a fact that indirectly emphasizes the anti-carcinogenic role of this element [67]. The different oxidation forms of selenium (selenium oxide, selenious acid, selenite salts) prevent: the formation of DNA adducts; DNA or chromosome breakage; and chromosome gain or loss, even on mitochondrial DNA. Preventing all the aforementioned genomic events improves the overall genomic stability [77]. A lesser known fact, but one that still supports genomic stability, is that selenium has also been linked to affecting telomere length and function [77]. The effects of selenium, selenium proteins and selenium binding proteins have been demonstrated clinically by several studies. Selenium binding protein 1 (SBP1) level has been correlated with lymph node metastasis and survival in the case of lung cancer [174]. This same protein has been demonstrated to have prognostic roles in nasopharyngeal carcinoma [175], breast cancer [176] and renal cancer [177]. More explicitly in breast cancer, SBP1 appears to regulate the antiproliferative effects of selenium [176].

The protective role of selenium in lung cancer has been demonstrated in a meta-analysis, presenting a decrease in cancer incidence with its consumption [178]. Selenium dietary levels were shown to be linked to selenoprotein expression, and to affect the immune response by influencing interferon-γ and IL-6 secretion [179]. TXNRD1, a selenoprotein, was shown to be overexpressed with a fold change of 1.5 in lung cancer compared to the adjacent normal tissue [180].

Selenium treatment was associated with reduced levels of mRNA for the DNA methyl transferases (DNMTs) 1 and 3A; moreover, this effect was further confirmed at the protein level for DNMT1 [75]. Selenium is able to restore the expression of hypermethylation-based silenced genes *GSTP1*, *APC* and *CSR1* in human prostate cancer cells by the downregulation of *DNMT* and inhibition of HDAC activity [181].

Identified two centuries ago by Berzelius, selenium is an essential element of life processes. Despite this research field flourishing in recent years, the role of most of the selenoproteins is still unclear [76]. It remains important to evaluate the complex role of selenium, in the context of its absorption, metabolism, and excretion capacity relative to individual selenoprotein genotypes. This can be analyzed using systems biology approaches, combining nutrigenetics and nutrigenomics for optimizing the implementation and real-time monitoring of selenium.

## 5. Polyunsaturated Fatty Acids (PUFAs)

Another highly debated topic related to cancer risk is represented by polyunsaturated fatty acids (PUFAs), which are essential for cellular homeostasis. Disruptions in their metabolism lead to cellular abnormalities and increased cancer risk. The production of unbalanced pro- and anti-inflammatory lipid metabolites can activate cell proliferation, angiogenesis, and migration [78]. Even if the current status of PUFAs is quite inconsistent regarding cancer, there is interest regarding the anti-carcinogenic properties of these molecules if administrated in correct doses, ratios and intervals. PUFAs such as ω-3 and ω-6, also known as ω-3 and ω-6 fatty acids, have an important effect on transcriptome expression patterns, not coincidentally related to lipid and carbohydrate metabolism. PUFAs also seem to be interconnected to two genetic polymorphisms, *APOA1*−75G→A and *PPARA* Leu162Val, having an effect on cardiovascular disease risk factors [182]. Firstly, increased PUFA intake, in patients with *APOA1*−75G→A polymorphism, decreased HDL-cholesterol concentrations without affecting triacylglycerol concentrations. Secondly, increased PUFA intake caused decreased triacylglycerol concentrations in patients, specifically with the *PPARA* Leu162Val polymorphism [182,183].

ω-3 and ω-6 fatty acids, or their specific metabolic products, are able to target a wide variety of key players in essential pathways: transcription factors like PPARs; nuclear factor κ-light-chain-enhancer of activated B cells (NFκB); or molecules related to inflammation such as tumor necrosis factor (TNFα), IL-1β or IL-6 [184]. Furthermore, PUFAs interfere with angiogenesis (VEGF, platelet derived growth factor-PDGF, MMP-2), cell cycle and proliferation (cyclins, p53, phosphate and tensin homolog-PTEN) molecules, all leading to the activation of tumorigenic pathways [79]. More specifically, ω-3 PUFAs have shown to contribute to the chemoprevention of oral cancer, by regulation via β-catenin signaling pathways [185] or via ERK1/2 phosphorylation [186]. In the end, one of the primary sources of lipid molecules is polyunsaturated fatty acids, representing the building blocks of the cell and its processes.

## 6. Prebiotics, Probiotics and Dietary Fibers

The human body includes a personalized microbiome that is indispensable for health support, but also capable of inducing pathological states [187]. The regulation of microflora composition offers the possibility of disease prevention through the control of the involvement of mucosal and systemic immunity [187]. There is very good rationale for the microbiota to be taken into consideration, when infections could account for 15% of all worldwide malignancies [188].

Probiotics are described as live microorganisms administered in suitable amounts, to give a health benefit to the host [189], meanwhile prebiotics are selective substrates used by host microorganisms, providing a health benefit [189]. These two systems are designed to revive the normal balance of gut microbiota [189].

The oral cavity microbiota is related to a wide range of oral diseases and cancer of the aero-digestive tract [190]. Understanding the relationship between microbiota and susceptibility towards oral carcinogenesis could guide new approaches using prophaylactics or new microbiota-enhancing therapies [190,191]. The strategy of preventing bacterial and viral infection to hinder the development of cancer could use oral cancer and oral cavity microbiota as a proof of concept. This notion is supported by the fact that infections from bacterium or viruses are associated with the incidence of certain cancers: the bacterium Helicobacter pylori has been casually correlated to gastric adenocarcinoma; Epstein–Barr virus was conclusively proven as a carcinogen for non-Hodgkin’s lymphoma, Hodgkin’s disease and nasopharyngeal carcinoma; lastly, Human Papillion virus increased association to cervical cancer [192].

There are three main mechanisms by which infections can cause cancer, primarily facilitating the initiation and promotion of carcinogenesis. Firstly, the infectious agent becomes persistent in the host, thereby inducing chronic inflammation. Secondly, infectious agents can directly transform cells by inserting active oncogenes into the host genome or by inhibiting tumor suppressor genes. Thirdly, infectious agents can induce immunosuppression and consequently reduce immunosurveillance [188]. Thus, preventing carcinogenesis through the use of microbiota needs to target at least one of these three mechanisms.

Studies have accumulated investigating the effect of prebiotics and probiotics consumption from fermented or unfermented dairy products on cancer, albeit indirect experimental evidence of cancer suppression in human patients. The recent in vitro and in vivo study results are promising, with an indication that probiotic bacteria reduce the risk, incidence and number of colon, liver or bladder tumors. This protective effect against cancer development can be ascribed to multiple general biological explanations: enhancing the immune system of the host, modulating oxidative stress and inflammation, or maintaining the healthy bacterial populations such that they outcompete/suppress bacteria that produce carcinogens. However, more specifically, probiotic intake is currently associated with the components from lactic bacteria capable of modulating immune response, principally by the regulation of several factors like interleukins (Interleukin-12) and tumor necrosis factors (TNFα), concurrently improving the cytokine-associated pathways [91,193,194].

On the other hand, prebiotics and gut microbiota are in direct relationship with a wide range of pathologies like obesity or inflammatory processes [195]. Both insoluble and soluble dietary fibers can affect the intestinal bastion’s absorption rate. Moreover, an extensive selection of xenobiotics are reported to be involved with cancer chemoprevention mechanisms [83]. For example, as presented in Table 1, for the cases of stomach and ovarian cancer, an inverse relationship was observed between cancer risk and various types of fibers derived from vegetables and fruits [85,86]. At the same time, breast cancer protection by dietary fibers was achieved either by blocking the intestinal absorption of estrogens released by biliary systems, or by modulating insulin-like growth factors and insulin resistance [81,82].

The gene exchanges within the gut microbiota were demonstrated to be more frequent than expected [196]. The protective action of dietary fibers is attributed to their ability to dilute toxic environmental agents and to increase the intestinal tract transit, therefore leading to a reduced absorption at the intestinal level. Dietary fiber supplementation brings physical changes in microbiota composition [195], and these changes involve horizontal gene transfer either through transduction or bacterial conjugation. This bacterial genetic crosstalk, in turn, improves human health from a meta-genomic perspective [195].

Last but not least, probiotic therapy offers an interesting approach to stimulate host health via the transportation of anti-inflammatory mediators [87]. The human gut microbiome is represented by a highly complex ecosystem of uncultured bacteria, responsible for the catabolism of dietary fibers that were not metabolized in the upper digestive tract due to a lack of carbohydrate active enzymes (CAZymes) [92]. Studies identified 33 CAZymes encoding genes with a high homologous structure, from a meta-genomic dataset consisting of at least 20 individuals. Furthermore, 18 multigenic clusters encoding complementary enzymes responsible for plant cell wall digestion have also been identified [92].

## 7. Conclusions and Further Perspectives

**Figure 2:**
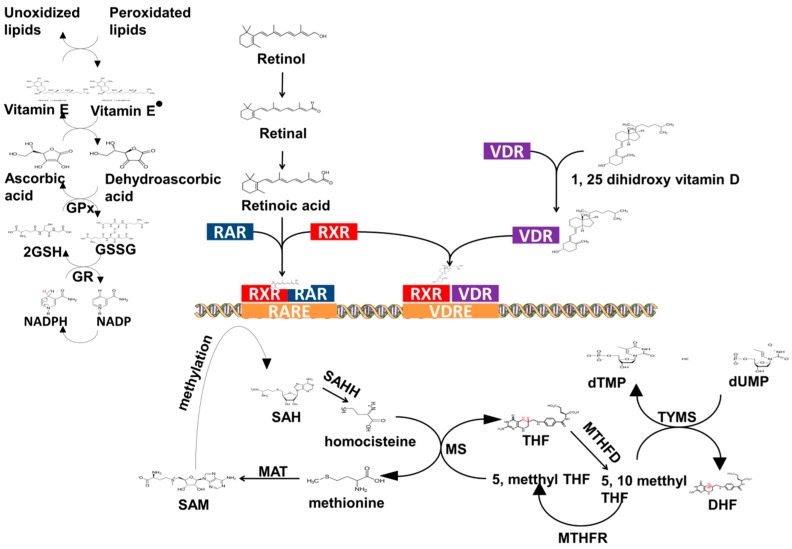
Pathways or interactions representative of the metabolism of nutrients. The majority of the nutrients function either as electron transporters in redox systems or as ligands for transcription factors involved in gene regulation. These effects can be intertwined, as in the case of folate metabolism. Folate metabolism has a dual effect in that it facilitates protein methylation by providing 1-carbon source influencing gene regulation, and it acts in the redox system of oxidative stress by influencing the levels of homocysteine. GPx = glutathione peroxidase; GSH = reduced glutathione; GSSG = oxidized glutathione; GR = gluthatione reductase; NADP = nicotinamide dinucleotide phosphate; RAR = retinoic acid receptor; RXR = retinoid X receptor; RARE = retinoic acid response element; VDR = vitamin D receptor; VDRE = vitamin D response element; dUMP = deoxy uridine monophosphate; dTMP = deoxythymidine monophosphate; TYMS = thymidilatesynthetase; DHF = dihydrofolate; T/HF = tetrahydrofolate; MTHFR = methylene tetrahydrofolate reductase; MTHFD = methylene tetrahydrofolate dehydrogenase; MS = methionine synthetase; SAM = S-adenosyl methionine; SAH = S-adenosine homocysteine; MAT = methionine adenosine transferase; SAHH = S-adenylhomocisteine hydrolase.

Despite the highly debatable role of natural compounds in combating cancer, these products are now emerging as important factors for cancer prevention or inhibition. Even if their activity is not necessarily directly correlated to the induction of cancer cell apoptosis, the intake of vitamins or other molecules from various food sources or synthesized drugs is starting to be more thoroughly investigated using next-gen technologies in oncology. Therefore, any deficiency in the previously mentioned nutrients has been correlated to a majority of cancers, and their genomic characterization can distinguish important information involving mechanisms and pathways. This complex biological effect can be deciphered using systems biology approaches, specifically evaluating the optimal dose of these micronutrients, in order to maximize the beneficial effects. Table 2 summarizes the impact that nutrients can have in cancer therapy, while the metabolism, including mechanism of action, of certain representative nutrients can be seen in Figure 2.

Considering the aforementioned facts, it is becoming clearer that one way to obtain optimal effects for health in general, and in cancer patients in particular, is to optimize diet for each individual, taking into account their metabolic requirements. As previously mentioned, this approach can be pursued through both nutrigenomics and nutrigenetics. By analyzing the potential genetic response of an individual to a set of nutrients, it will be possible to recommend an ideal treatment diet that synergistically works as an adjuvant in the inhibition of processes associated to specific malignancies. As time passes, it will become more about personalized nutrition and less about one-size-fits-all “good” diets; moreover, detailing the “good or bad” quantities of a certain nutrient. It should be remembered that diet alone cannot work in preventing or treating cancer, but should always be seen as an irremovable part of the whole array of molecular interactions that determine individual health. As presented in this review, there is a lot of accumulated data regarding nutrients yet to be analyzed and integrated into the bigger picture of personalized medicine. In addition, there is a dire need for an integrated multi-omic strategy incorporating nutrients and health, in order to obtain patient-specific beneficial outcomes concerning disease.

## Figures and Tables

**Table 1:** List of techniques presently utilized at each “omics” level (DNA, RNA, protein, metabolite) that could determine the impact of nutrients on human health, with emphasis on the practical application.

	Nutrigenetics	Nutrigenomics	Practical Application	Ref.
**DNA**	Next generation sequencing (NGS), pyrosequencing, nanostring, polymerase chain reaction (PCR)-based methods	Microarray, NGS, nanostring	Methods assessing DNA are more prone to be applied in nutrigenetics, with emphasis on particular mutations or single nucleotide polymorphisms (SNPs) that affect the response to a particular diet. This entails prediction of genotype/mutation patterns caused by the indirect interaction of genes with certain nutrients.	[14,15,16,17,18,19,20,21,22]
**Coding and non-coding RNA**	Next generation sequencing, pyrosequencing, PCR-based methods	Microarray, NGS, nanostring	Methods assessing RNA are more prone to be applied in nutrigenomics, to evaluate the effect on the alteration of coding and non-coding genes of a particular nutrient. This means determining RNA levels from different tissues to observe the effects of nutrients on transcriptomic profile in terms of impact on physiological or pathological status.	[23,24,25,26,27,28]
**Proteins**	Mass spectrometry (MS), high performance liquid chromatography (HPLC), high performance liquid chromatography–tandem mass spectrometry (HPLC/MS), ultra-high performance liquid chromatography–tandem mass spectrometry (UHPLC/MS)	HPLC/MS, UHPLC/MS	Proteomics is also more prone to be found in nutrigenomic studies. Being an extension of transcriptomics, it allows for validating mRNA expression protein levels.	[28,29,30,31]
**Metabolites**	Nuclear magnetic resonance, HPLC/MS, UHPLC/MS	Nuclear magnetic resonance, HPLC/MS, ultra-high performance liquid chromatography (UHPLC)	Giving a complete picture, metabolites are able to be more accurate in predicting the effect of nutrients. Furthermore, they could be used for validation of the other “omics.”	[22,32,33,34,35,36]

**Table 2:** List of experimentally investigated nutrients with a potential impact on cancer therapy, determined by cancer type, expected outcomes and genes effected.

Nutrient	Cancer type	Expected Outcomes	Genes effected	Comment	Ref.
**Vitamin A**	Glioma, lung, colorectal cancer	Pro/anti-oxidant action, cell differentiation and immune response	Expression level and polymorphism of RARs, RXRs, and PPARβ/δ, Akt, Erk, JNK, p38	Epidemiological data are not consistent	[51,53,54,55,56]
**Vitamin C**	Solid tumors and hematological malignancies	Selective activation of apoptosis and autophagy. Interferes with redox-sensitive transcription factors and associated target molecules. Selective metabolic and genotoxic stress on tumor cells.	Expression level and polymorphism of GLUT, GST, MnSOD, SVCT, Hp	Low toxicity to normal tissues, but with controversial data due to its dual effect as a pro/antioxidant. The molecular mechanism(s) of selective toxicity on tumor cells remains to be deciphered	[40,49,57,58,59,60,61,62]
**Vitamin D**	Colorectal, breast, prostate or pancreatic cancer	Correlated with lower risks of specific cancers.	Expression level and polymorphism of VDR target genes like p21^WAF1/CIP^ TP53, p27, Cyclin C, *CYP24* gene	The results of these studies have been inconsistent, possibly because of the challenges in carrying out such studies.	[63,64,65,66]
**Vitamin E**	Prostate, breast colorectal cancer	Reduces unwanted side effect of cytotoxicity by targeting oxidative stress and inflammatory markers	Polymorphism of *APOA5, CYP4F2*	This might also have a pro-oxidant effect.	[51,57,67,68,69,70]
**Folic acid**	Gastric colorectal, breast, pancreatic cancer	Carcinogenesis and embryonic development. At low doses, it decreases cancer risk but overdoses might increase cancer risk	Methylation of *DIRAS3*, *ARMC8*, *NODAL, MTHFR* and HOX genes	Dual role: protection early in carcinogenesis and at high doses in late stages of cancer	[71,72,73]
**Selenium**	Prostate, breast, lung, oropharyngeal, colorectal, bladder, skin, leukemias, uterine, ovarian cancers	Antioxidant, reduces cancer risk; restores epigenetic altered events; genomic stability	Expression and polymorphism of GPxsang, TrxRs	Still highly controversial, being tumor specific and dose specific (pro/antioxidant effect)	[63,67,74,75,76,77]
Polyunsaturated fatty acids (**PUFAs**)	Breast, colorectal cancer	Regulate cytokine production; stimulate the immune response and enhances apoptosis in cancer cells; regulate cell proliferation and angiogenesis	Transcription factors: PPARs or NFκβ; immune response: TNFα, IL-1β, IL-6; angiogenesis mechanisms: VEGF, PDGF, MMP-2; cell proliferation: cyclins, p53, PTEN	Involved in tumor biology and cancer patients’ prognosis; epidemiologic data furnish inconsistent picture	[63,78,79,80]
**Dietary fibers**	Colorectal, breast, pancreatic, ovarian or stomach cancer	Increased intestinal transit blocking the absorption of external or internal toxic factors	Expression level and polymorphism of CAZymes family	Highly controversial epidemiological data, due to the different types of soluble or insoluble fibers used in studies	[81,82,83,84,85,86,87,88,89,90]
**Probiotics**	Colorectal cancer	Cell-mediated immune responses; increase the activity of antioxidant enzymes	Expression level and polymorphism of CAZymes family	Presently there is no direct evidence in epidemiological data	[87,91,92,93,94,95]
